# Disproportionate Impacts of Wildfires among Elderly and Low-Income Communities in California from 2000–2020

**DOI:** 10.3390/ijerph18083921

**Published:** 2021-04-08

**Authors:** Shahir Masri, Erica Scaduto, Yufang Jin, Jun Wu

**Affiliations:** 1 Department of Environmental and Occupational Health, Program in Public Health, University of California, Irvine, CA 92697, USA; masris@uci.edu; 2 Department of Land, Air, and Water Resources, University of California, Davis, CA 95616, USA; escaduto@ucdavis.edu (E.S.); yujin@ucdavis.edu (Y.J.)

**Keywords:** wildfire, environmental justice, climate change

## Abstract

Wildfires can be detrimental to urban and rural communities, causing impacts in the form of psychological stress, direct physical injury, and smoke-related morbidity and mortality. This study examined the area burned by wildfires over the entire state of California from the years 2000 to 2020 in order to quantify and identify whether burned area and fire frequency differed across Census tracts according to socioeconomic indicators over time. Wildfire data were obtained from the California Fire and Resource Assessment Program (FRAP) and National Interagency Fire Center (NIFC), while demographic data were obtained from the American Community Survey. Results showed a doubling in the number of Census tracts that experienced major wildfires and a near doubling in the number of people residing in wildfire-impacted Census tracts, mostly due to an over 23,000 acre/year increase in the area burned by wildfires over the last two decades. Census tracts with a higher fire frequency and burned area had lower proportions of minority groups on average. However, when considering Native American populations, a greater proportion resided in highly impacted Census tracts. Such Census tracts also had higher proportions of older residents. In general, high-impact Census tracts tended to have higher proportions of low-income residents and lower proportions of high-income residents, as well as lower median household incomes and home values. These findings are important to policymakers and state agencies as it relates to environmental justice and the allocation of resources before, during, and after wildfires in the state of California.

## 1. Introduction

Since 1980, the United States (US) National Oceanic and Atmospheric Administration has reported an approximately threefold increase in so-called “billion-dollar” natural disasters across the United States [1]. Catastrophic wildfires with over one billion dollars in losses have increased from an average of 1.5 events per decade from 1980–1999 to seven per decade from 2000–2019, costing the nation a cumulative $10 billion and $75 billion (USD) over these two time periods, respectively [2]. Health-related costs from smoke exposure are also estimated in the billions of dollars [3]. The western US tends to experience the majority of the nation’s wildfire burning (in terms of acreage) due in part to its dry climate [4]. However, wildfires in this region have grown more severe and extensive over time, with one study showing a fourfold increase in the occurrence of major wildfires, a six-fold increase in the area burned by such fires, an increase in average fire duration from 7.5 to 37.1 days, and a 78 day increase in the length of the wildfire season between 1987 and 2003 relative to the 1970–1986 average [4].

In California, along with the neighboring states of Oregon and Washington [5], the last 5 years have seen a historic increase in wildfire activity. Of the top 20 largest wildfires recorded in California, over half occurred in the last decade, with five of the six largest occurring in just 2 months in 2020 [6]. In 2017, the Tubbs Fire became the most destructive wildfire on state record, claiming 5,636 structures and 22 lives [7]. Two months later, the Thomas Fire became the state’s largest and latest burning wildfire (not contained until January) [6]. In 2018, California broke wildfire records again as the Mendocino Complex Fire and Camp Fire became the new “largest” and “most destructive” wildfires on record, respectively; the latter killed 85 people in the city of Paradise, making it the deadliest wildfire in state history by nearly threefold [8]. As of 2020, the August Complex Fire (>1 million acres) is now California’s largest wildfire, more than doubling and quintupling the former largest wildfires which occurred in 2018 (Mendocino Complex Fire: 459,123 acres) and 2017 (Thomas Fire: 281,893 acres), respectively [6]. The California Department of Forestry and Fire Protection (CAL FIRE), the state’s primary fire agency, highlights climate change as a factor contributing to the exacerbation of wildfires [9], as average temperatures in California have steadily increased since the early 1900s [10]. As drought conditions and extreme winds become more frequent and vegetation more dry, the risk of major wildfires is projected to increase [9,11].

These massive wildfires cause serious health impacts, ranging from air pollution impacts to fire-related mortality and mental stress [12,13,14,15,16]. During wildfire episodes, smoke often results in two- to fourfold increases in concentrations of particulate matter with an aerodynamic diameter < 2.5 μm (PM_2.5_), a form of air pollution widely associated with respiratory disease and cardiovascular disease, as well as all-cause mortality and hospital admissions [13,17,18]. PM_2.5_ levels exceeding the 24 h national standard of 35 μg/m^3^ have been reported during wildfire events in southern California, with concentrations in some cases over 10 times (>230 μg/m^3^) higher than background levels [19,20]. Importantly, wildfire impacts to individuals may be exacerbated depending on the socioeconomic characteristics of affected communities [14,21].

Currently, individuals in the US are at a greater risk of being impacted by a wildfire compared to earlier periods in history due to continued encroachment of human settlements into the wilderness and, thus, expansion of the wildland–urban interface (WUI). The WUI has been recognized as an area highly prone to wildfires, mostly due to a higher human-caused ignition probability, mixed fuels, greater vegetation, and distance from firefighting resources [9,22,23]. As of 2010, 33% of US housing units were within the WUI, while 43% of new housing built across the country from 1990 to 2010 was within the WUI [24,25]. In California, the percentage of homes within the WUI is very similar (32.4% as of 2010), amounting to 11 million people inhabiting 4.4 million housing units within the WUI, up one-third compared to 1990 [24,25].

As wildfires become more widespread and their impacts more severe, it is important to understand which communities are being affected and how this differs in terms of socioeconomic characteristics. To date, findings regarding wildfire impacts and community risk have been mixed. In the upper Midwest, areas with less frequent wildfire occurrences have been shown to consist of a greater proportion of owner-occupied housing units, as opposed to renter-occupied units [12]. Similarly, in a study examining the US, Census tracts that were majority Black, Hispanic, or Native American were 50% more vulnerable to wildfire compared to other Census tracts [26]. In contrast, Wigtil et al. (2016) examined the overlap between wildfire potential and social vulnerability across the contiguous US and found that, on average, places with high wildfire potential have lower social vulnerability than other places [27]. While numerous studies have examined wildfire hazard potential in the United States both within and outside the context of socioeconomic vulnerability, studies mainly focus on aggregated vulnerability indicators and vulnerability indices, which do not identify which individual vulnerability indicators actually correlate with historic burning [24,26,27,28]. Furthermore, studies that considered socioeconomic characteristics typically examined wildfire hazard potential as opposed to actual fire occurrence.

In this study, we examined the area burned by wildfires over the entire state of California from the years 2000 to 2020 in order to understand how wildfire activity has changed over time and to identify how impacts have varied according to individual socioeconomic factors at the Census tract level. Specifically, our research questions were as follows: (1) How have changes in the number of wildfires and area burned by wildfires in California changed since 2000? (2) Given changes in wildfire activity in California, how has the composition and number of impacted people changed over time? (3) Is the area burned by wildfires correlated with socioeconomic factors at the Census tract level, and does this correlation differ between urban and rural areas? (4) How did the record-breaking 2020 wildfire season affect the trend in overall wildfire activity and the correlation between wildfire-burned area and socioeconomic vulnerability?

We hypothesized a growth trend in wildfire activity that disproportionately impacted Census tracts of greater socioeconomic vulnerability in California. We also hypothesized that urban/rural designations would influence the relationship between wildfire activity and socioeconomic factors such that rural areas would exhibit stronger positive correlations with wildfire activity. Collectively, this study contributes to the literature by advancing our understanding of the wildfire impacts sustained among communities of disproportionate physical and socioeconomic vulnerability, in turn highlighting areas and populations in need of more focused municipal aid and recovery efforts before, during, and after major wildfire events.

## 2. Materials and Methods

### 2.1. Wildfire Data

This study examined the area burned by wildfires across the entire state of California, USA, over a period of 21 years (2000–2020) using fire perimeter data compiled from the California Fire and Resource Assessment Program (FRAP) and National Interagency Fire Center (NIFC). These agencies estimate the area burned by wildfires according to different methods and protocols, which can result in many nonoverlapping burned areas. For instance, FRAP consolidates fire perimeters from multiple agencies including CAL FIRE, the US Forest Service, Bureau of Land Management, and National Park Service on the basis of wildfire perimeter availability and size requirements, and they are updated once annually. NIFC pools wildfire data similarly, but also makes use of the US Department of Agriculture Forest Service’s National Infrared Operations (NIROPS) program, which determines fire perimeters primarily using thermal infrared imaging from nighttime airborne flights [29]. These perimeters are created to assist fire management efforts and are calculated using different methods (e.g., aircraft, global positioning system handheld devices, and/or manual digitizing, post-processing), depending on the availability of resources.

In order to obtain complete data relating to the area burned by wildfires across California and to avoid having missed any reported wildfires, we extracted all wildfire perimeter data across both FRAP and NIFC sources spanning the years 2000 to 2020. FRAP data were obtained by visiting the FRAP website (https://frap.fire.ca.gov/mapping/gis-data/, accessed on 2 April 2021) and selecting the “Fire Perimeters” tab, where data can be directly downloaded, while NIFC data were similarly obtained by visiting the open data section of the NIFC website (https://data-nifc.opendata.arcgis.com/search, accessed on 2 April 2021) and downloading the data available in the files titled “Historic Perimeter Combined 2000–2018”, “Historic Perimeters 2019”, and “Operational Data Archive 2020”. Links to these webpages are provided in the data availability statement at the end of this manuscript. After extracting all wildfire perimeter data, we pooled all wildfire records together in ArcGIS (ESRI, Redlands, CA, USA). To avoid double counting, all duplicate wildfires were then removed during data processing. Given that a minority of Census tracts (<1%) had more than 100% of their area burned by multiple fires over the 21-year study period, our calculation of total wildfire area may reflect a slight underestimation of true burned area over this period.

### 2.2. Demographic Data

This study used the most recently available (2010) Census data to obtain population counts for all Census tracts (*n* = 8057) across the state of California. The American Community Survey (ACS), conducted every year, was also used to obtain information about household income, race/ethnicity, education, insurance coverage, age, and other socioeconomic characteristics at the Census tract level. For the ACS, 5-year averaged data from 2018 were used since averages provide a more stable representation of community-level factors, and because 2018 was the most recent year for which 5-year average data were available, whereby wildfire burned area was greatest in the more recent, as opposed to earlier, time period of the study. ACS data were not utilized across multiple years of the study period so as to eliminate potential changes in social and economic characteristics that may occur slightly from year to year, particularly after a major wildfire, as well as characteristics such as population size, home values, and income, which tend to exhibit positive trends over time irrespective of wildfire activity.

### 2.3. Analysis

Annual burned area and cumulative (all 21 years) burned area were calculated across the state of California and for each Census tract in California from 2000 to 2020 using ArcGIS software (ESRI, Redlands, CA, USA). To understand the overall trend in wildfire activity over the 21-year period, annual and cumulative wildfire area were plotted in the form of a histogram. We then overlaid the Census tract layer and wildfire perimeters to identify Census tracts that were affected by wildfires during a given year. The analysis examined trends in the number of Census tracts that incurred different percentages of burned area (>0%, >10%, >25%, >50%, and >75%), the number of Census tracts burned by wildfires of varying sizes (any size, >100 acres, >1000 acres, >10,000 acres), and the total size of the populations residing in Census tracts affected by wildfire each year from 2000–2020. These categories were not mutually exclusive (e.g., “>100 acres” category included fires in the “>1000 acres” category). Census tracts considered to be affected by wildfire were those where at least one wildfire took place during a given year.

Summary statistics of socioeconomic characteristics were calculated for each Census tract across the state. The study focused on the Census tract-level distribution of the percentage of socioeconomic characteristics that could reasonably be expected to render a community at increased vulnerability to wildfire, including female and male residents, residents who identified as Hispanic, Asian, African American, or Native American, immigrant non-native residents (henceforth immigrants), residents who reported speaking no or limited English, residents who did not have health insurance coverage, residents under 5 years of age and those over 65 years of age, renter-occupied housing units, vacant housing units, residents who used public transportation, residents with a college education or higher, and whether or not households had computers and internet. We also calculated statistics relating to economic factors such as unemployment rate, poverty rate, median household income, and median home value.

We first calculated summary statistics for socioeconomic factors across Census tracts that were grouped according to the number of years that they experienced at least one wildfire (henceforth “fire frequency”). Fire frequency for a given Census tract reflects how often it experienced direct wildfire, with values ranging from a minimum of 0 (indicating no years with wildfire during the study period) to 21 (indicating wildfire every single year during the 21-year study period). Socioeconomic factors were compared across each fire frequency category using scatter plots with accompanying trendlines.

Given that Census tracts with larger areas are more likely to experience wildfires (all other factors being equal), thus having higher values of fire frequency, this analysis further evaluated the relationships between Census tract socioeconomic characteristics and wildfire impacts by the percentage of cumulative burned area over the study period: 0% (no wildfire), 1–10%, 11–20%, 21–30%, 31–40%, 41–50%, 51–60%, 61–70%, and ≥71% of land area burned by wildfire. Linear trendlines were fit to help show correlations in either the positive or negative direction. By considering wildfire burned area on an annual basis, this study was able to account for overlapping burned areas that may have occurred during different years.

In describing our analysis of fire frequency and burned area, it is useful to identify the tradeoffs in terms of results. Fire frequency can help identify Census tracts that were repeatedly afflicted by wildfire over the study period. However, this metric may be influenced by Census tract size as previously mentioned. In contrast, burned area as a fraction of total Census tract area can overcome issues of Census tract size; however, it may bias in the opposite direction, since smaller Census tracts require smaller wildfires (which are more frequent) to produce the same fraction of burned area. Additionally, Census tracts that experienced major wildfires that burned large fractions of land area during the first half of the study period may be less likely to experience wildfires in subsequent years due to reduced fuel availability (e.g., forest no longer exists). The tradeoffs associated with each method reinforced our decision to employ both techniques when conducting this analysis. Census tracts that experienced a fire frequency greater than 10 (a fire at least every other year) and over 70% cumulative wildfire-burned area over the study period were defined as “highly impacted” and were compared with the group consisting of all other Census tracts in terms of their socioeconomic characteristics.

This study further evaluated whether correlations differed between urban and rural areas by separating Census tracts into the urban/rural designations of their zip codes based on rural–urban commuting area code (RUCA) approximations produced by the University of Washington’s Rural Health Research Center [30]. RUCA values ≤ 3 were considered urban, while values > 3 were considered rural. An example of a major California wildfire (the Thomas Fire) that took place from December 2017 to January 2018 is presented in the form of satellite imagery in Figure S1 (Supplementary Materials).

## 3. Results

### 3.1. Wildfire Activity Patterns and Trends

In total, 1081 of California’s 8057 Census tracts, housing 5.3 million (13.4%) of the state’s 39.5 million residents, experienced wildfires from 2000 to 2020. Of those, 694 experienced wildfires during at least two separate years over the study period, amounting to 8.6% of total Census tracts housing 3.4 million people across the state. Of the Census tracts that experienced wildfires, 358 Census tracts housing 1.7 million residents and 218 housing 0.9 million residents saw at least 25% and 50% of their land area burned by wildfires over the course of the study period, respectively. There were 46 Census tracts housing roughly 216,000 residents that experienced an amount of wildfire burning equivalent to 100% or more (max = 187%) of the total area of the Census tract.

Our results showed 16.7 million acres of land burned by wildfires in California from 2000 to 2020, which corresponds to 15.9% of the 105 million acres of the state’s total land area. Of this total, 43.7% occurred over just three wildfire years (i.e., 2008, 2018, and 2020), while 61.8% occurred over the six largest wildfire years (i.e., 2003, 2007, 2008, 2017, 2018, and 2020). Annual area burned by wildfires across the state was highly correlated (*r* ≥ 0.7) with the number of Census tracts annually burned by wildfires that were ≥1000 acres in size, and moderately correlated with the number of Census tracts that experienced >10% and >25% of annual area burned. A complete correlation matrix showing the temporal relationship between total annual burned area and other key wildfire statistics is presented in the Supplementary Materials (Table S1).

**Figure 1:**
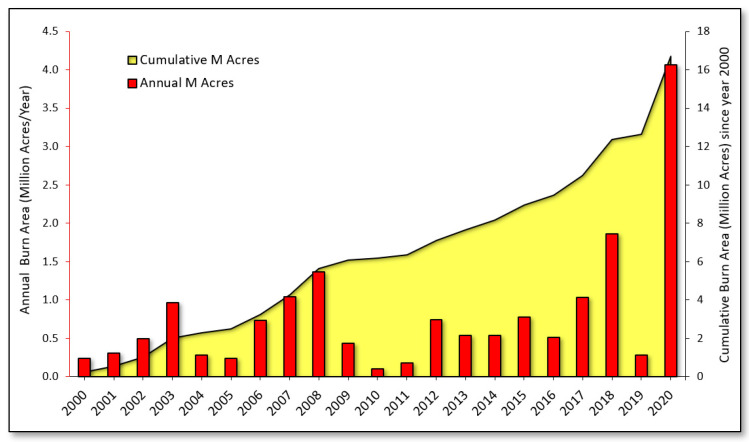
Cumulative and annual wildfire-burned area in California from 2000–2020.

Figure 1 presents annual and cumulative wildfire-burned area across the state of California from 2000 to 2020. It shows a trend of increasing burned area over time, with an 8.2% increase in wildfire-burned area that occurred in the second decade of the study period (2010–2019) relative to the first (2000–2009). Year 2020 was a record-breaking wildfire year for the state, resulting in over 4 million acres burned. This amount corresponds to roughly one-third of the area burned by each preceding decade. When the analysis included 2020 burned area in a comparison with the first (2000–2009) and last (2011–2020) decades of the study period, we observed a 73% increase in burned area in the last decade.

The linear trend demonstrated a 64,713 acre/year increase in wildfire-burned area, resulting in an approximately 10-fold increase in area burned from the year 2000 to 2020. This trendline was heavily influenced by the record-breaking 2020 wildfire season. When 2020 wildfire data were excluded, the slope of the trend decreased substantially, albeit still resulting in a 23,254 acre/year increase and 2.4-fold overall increase in area burned over two decades.

### 3.2. Census Tract Impacts

**Figure 2:**
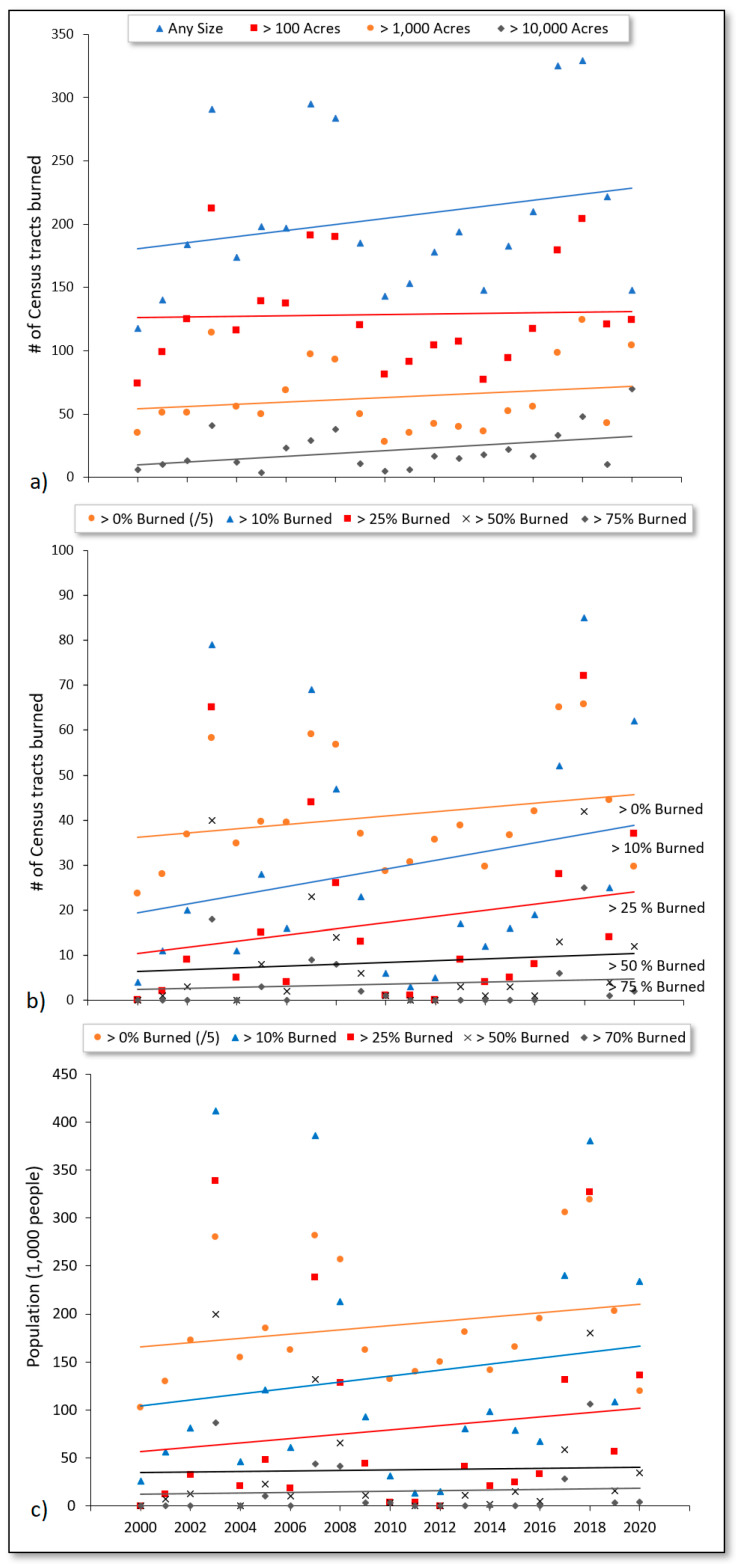
Annual time series of the (**a**) number of Census tracts that incurred different percentages of burned area, (**b**) number of Census tracts burned by wildfires of varying sizes, and (**c**) cumulative population of those residing in Census tracts affected by wildfires from 2000–2020. For the Census tract “>0% burned” category, *y*-axis values were divided by 5 to adjust for differences in scale and enable a side-by-side comparison of trends.

Results generally showed that a greater number of Census tracts sustained wildfire burning over time across all wildfire sizes assessed, accompanied by a greater number of impacted residents. Overall, the number of Census tracts burned by wildfires showed a positive temporal trend (*r* = 0.04 to 0.42) during the past two decades, when grouped by wildfires of varying sizes (Figure 2a) and different percentages of burned area (Figure 2b) each year from 2000–2020. Similarly, an annual increase in the total population residing in fire-affected Census tracts was found over time. The annual number of Census tracts impacted by wildfires of any size increased from approximately 180 to 230 (~28% growth) over the study period (Figure 2a). A smaller increase was found when considering the number of Census tracts impacted by larger wildfires (e.g., >100 acres).

In Figure 2b, the slope was steepest (slope; 0.5 to 1.0) when considering Census tracts with >0%, >10%, and >25% area burned compared to Census tracts with higher fractions (>50% and >75%) of area burned (slope < 0.5). When considering Census tracts with over 10% of their area burned, the linear trend (Figure 2a) showed a doubling in the number of Census tracts in this category over the 21-year study period, from approximately 20 to 40 Census tracts.

Figure 2c presents the cumulative population of those residing in Census tracts affected by wildfire each year from 2000–2020. As with Figure 2b, the trendlines in Figure 2c illustrate that the sharpest increases (slope > 2000) in the number of people affected by wildfire over time were apparent among Census tracts that sustained less burn area as a percentage of total area. When considering Census tracts with greater than 10% of their area burned, the linear trend in Figure 2c shows an increase in the annual number of affected residents, from approximately 100,000 to 170,000 people (70% increase). Census tracts with greater than 25% of their area burned showed an increase from approximately 55,000 to 100,000 residents affected annually (~82% increase).

### 3.3. Wildfire Impacts among Vulnerable Populations

**Figure 3:**
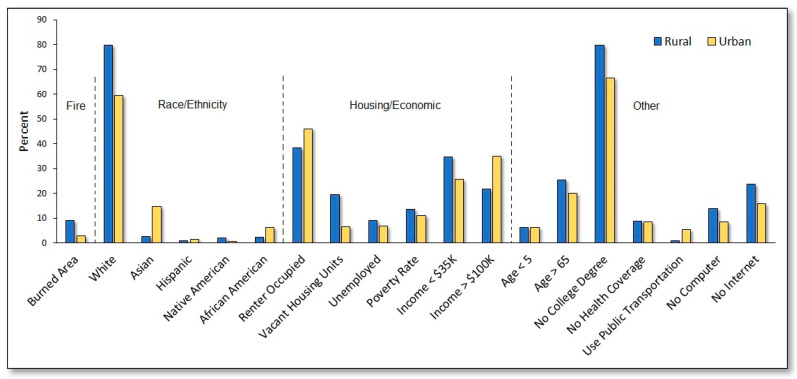
Wildfire vulnerability characteristics presented as percentages averaged across urban and rural Census tracts.

Figure 3 presents wildfire vulnerability characteristics as percentages averaged across urban and rural Census tracts, showing that rural areas were generally characterized by greater wildfire-burned area and lower socioeconomic status. Specifically, the wildfire-burned area average over the entire study period was over three times greater for rural compared to urban Census tracts. On average, rural areas also tended to have a higher proportion of White and Native American residents and lower proportions of Asian, African American, and Hispanic residents. Rural Census tracts also tended to have a higher proportion of households without computers, internet, use of public transportation, and median incomes < $30,000/year (henceforth “low-income residents”), as well as residents over age 65. In contrast, urban Census tracts tended to have higher proportions of households with a median income > $100,000/year (henceforth “high-income residents”), as well as lower rates of unemployment, poverty, and vacant housing units. The proportion of residents under age 5 and those without health coverage was approximately the same between urban and rural Census tracts.

**Figure 4:**
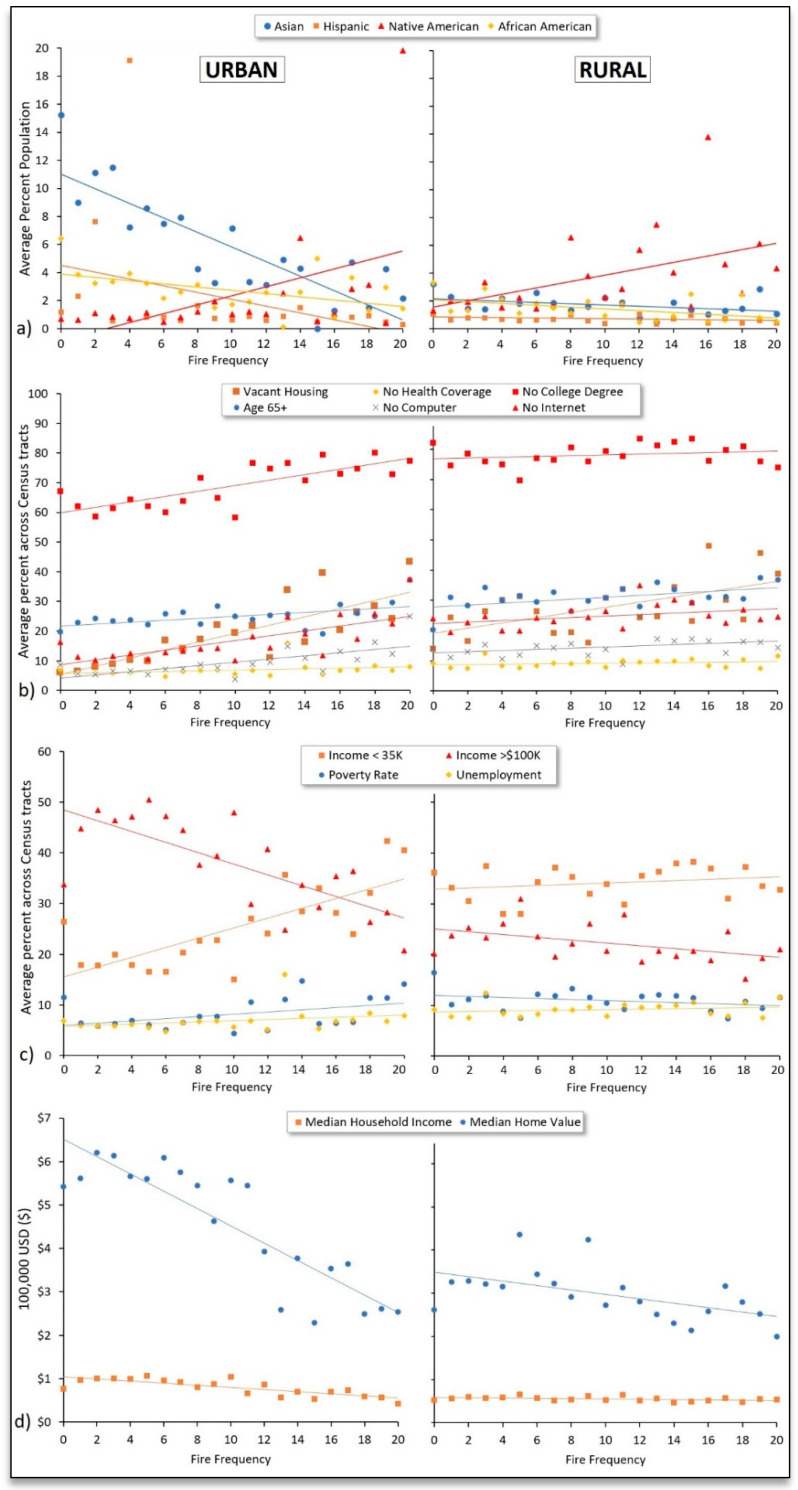
Percentage of (**a**) minority populations, (**b**) wildfire vulnerability indicators, and (**c**,**d**) economic indicators averaged across Census tracts grouped by fire frequency.

Figure 4 presents the percentage of minority populations and socioeconomic characteristics as a function of fire frequency (number of years), separately for urban and rural Census tracts, showing that wildfires were generally more frequent among areas that were more socioeconomically disadvantaged. On average, the proportion of minority populations was negatively correlated (urban, *r* = −0.84; rural, *r* = −0.36) with fire frequency (Figure 4a) except for Native American residents, of which there tended to be a higher proportion in Census tracts that had a higher fire frequency over the study period (*r* = 0.47 to 0.49). Although the percentages of African American, Asian, and Hispanic residents tended to be higher in urban areas (the opposite for Native Americans), trendlines exhibited the same directions between urban and rural areas. Although not shown in Figure 4, trendlines were similar for residents who identified as Hispanic, Asian, African American, or Native American, as well as for immigrant residents who reported speaking no or limited English. There was no trend as it relates to female/male differences.

Figure 4b illustrates increased wildfire vulnerability among Census tracts that experienced a higher fire frequency over the 21-year study period as indicated by higher proportions of older residents (age > 65) and residents/households without health coverage, a college education, occupancy, internet, and computers among Census tracts with a higher fire frequency. In urban areas, there was an approximately 50% increase in the proportion of older residents living in Census tracts with the greatest fire frequency compared to Census tracts with the lowest fire frequency, while, in rural areas, the Census tracts with the greatest fire frequency had a roughly 85% higher proportion of older residents. In general, urban Census tracts tended to exhibit steeper trendlines (i.e., slopes deviating from zero), particularly when considering vacant housing units and residents without a college degree. For instance, in urban areas, there was a 28% increase in the proportion of residents without a college degree living in Census tracts with the highest fire frequency compared to the lowest fire frequency, while the percentage increase was much more modest (~5%) when considering rural Census tracts.

Greater vulnerability among Census tracts with a higher fire frequency was also apparent for economic characteristics presented in Figure 4c,d, as reflected by higher proportions of low-income residents (*r* = 0.24 to 0.76), unemployed residents (*r* = 0.23 to 0.3), and residents living in poverty (urban only, *r* = 0.44), lower proportions of high-income residents (*r* = −0.74 to −0.47), lower median household incomes (*r* = −0.79 to −0.40), and lower home values on average (*r* = −0.88 to −0.54). Similar to Figure 4b, correlations tended to be higher in urban compared to rural areas.

**Figure 5:**
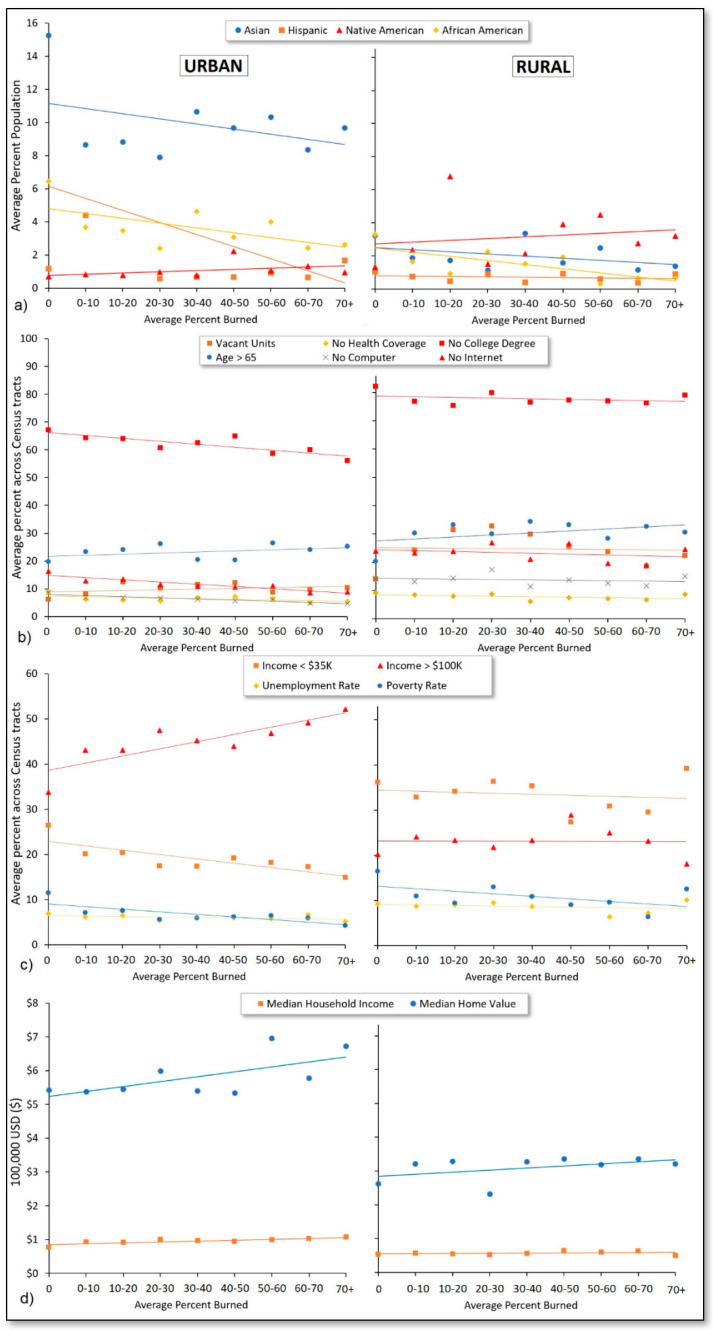
Percentage of (**a**) minority populations, (**b**) wildfire vulnerability indicators, and (**c**,**d**) economic indicators averaged across Census tracts grouped by the percent of cumulative Census tract land area burned from 2000–2020.

Figure 5 presents the percentage of minority populations and socioeconomic characteristics averaged across Census tracts grouped according to the percent of Census tract land area burned each year from 2000 to 2020 and by urban and rural areas. Results generally showed that socioeconomically advantaged Census tracts tended to see greater fractions of their land area burned by wildfire, relative to disadvantaged areas. Specifically, as with Figure 4a, Figure 5a shows the proportion of minority populations to be negatively correlated (*r* = −0.26 to −0.73) with fire burn area experienced by Census tracts on average. The exception again was for Native American residents (*r* = 0.18 to 0.41), of which there was a higher proportion in Census tracts that saw higher fractions of their land areas burned.

In Figure 5b, we observed consistency with Figure 4b in that older residents (age > 65) were in greater proportion in Census tracts that experienced a higher fraction of land area burning over the 21-year study period (*r* = 0.41 to 0.45). However, we also observed correlations that were more modest and, in some cases, opposite compared to those in Figure 4b. For example, the proportions of residents/households without a college degree, a computer, and internet were negatively correlated (*r* = −0.92 to −0.84) with the proportions of area burned in urban Census tracts. Unemployment rate exhibited little to no change across areas of different burn proportion in both urban and rural analyses, as did the proportion of residents without a college degree, households without a computer, and vacant housing units in rural areas.

When examining economic characteristics of Census tracts as a function of percent land area burned, urban Census tracts similarly exhibited patterns that were opposite those of Figure 4c, with lower proportions of low-income residents (*r* = −0.81) and higher proportions of high-income residents (*r* = 0.84) in Census tracts that experienced higher fractions of land area burning. For rural Census tracts, income exhibited little to no correlation with the proportion of burn area (*r* = −0.17 to −0.01). Similarly, in Figure 5d, urban Census tracts exhibited positive correlations when plotting average median household incomes (*r* = 0.84) and home values (*r* = 0.65) against the proportion of land area burned, whereas rural Census tracts showed positive albeit weaker correlations with these variables (*r* = 0.27 to 0.46).

**Figure 6:**
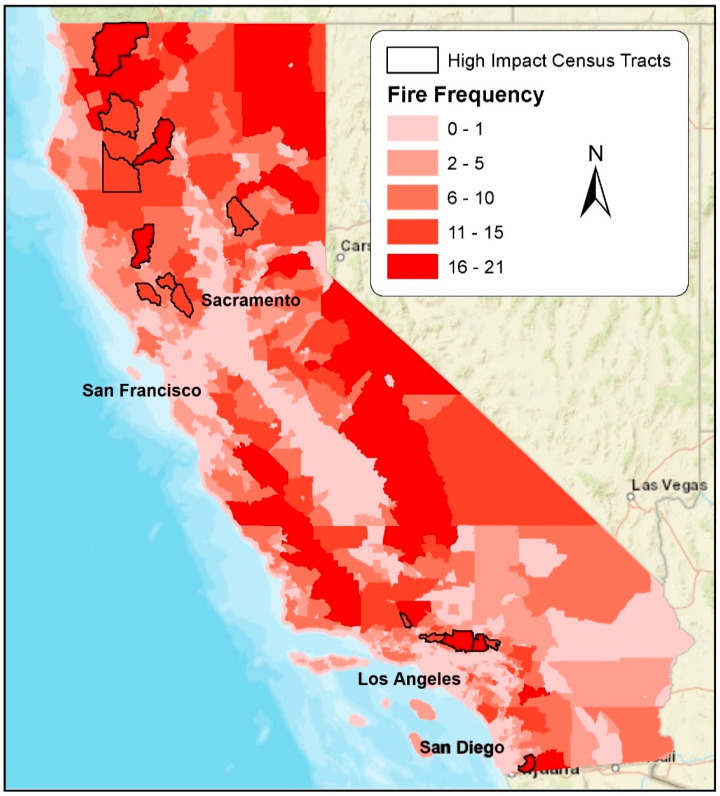
Number of years a Census tract experienced wildfire (fire frequency), along with 15 “highly impacted” Census tracts as defined by fire frequency and percent burning.

Census tracts that met our definition of being highly impacted according to both fire frequency and cumulative burned area included nine urban and six rural Census tracts. Relative to all other Census tracts, these 15 highly impacted Census tracts on average had lower proportions of minority group populations (except for the proportion of Native Americans which was over three times higher), as well as a 32%, 12%, 32%, and 36% higher proportion of older aged residents, residents without a college degree, households without internet, and households without a computer, respectively. These Census tracts also had 36% higher unemployment, with a 26% higher proportion of low-income residents, 17% lower proportion of high-income residents, 26% lower median home values, and 23% lower median household income relative to other Census tracts, on average. These trends were generally in the same direction for both rural and urban Census tracts, although disparities tended to be more dramatic among rural Census tracts. Highly impacted Census tracts, along with the fire frequency for all Census tracts, in California are presented in Figure 6 (using World Geodetic System of 1984, or WGS 1984).

The general findings presented in this section did not change when the analysis considered the first decade (2000 to 2009) and second decade (2010 to 2019) of our study period separately, nor when the record-breaking wildfire year of 2020 was either included or excluded.

## 4. Discussion

This study examined wildfire-burned area over the entire state of California from the years 2000 to 2020 in order to understand how wildfire activity has changed over time and to identify whether burned area and frequency differed across Census tracts according to socioeconomic characteristics over time.

### 4.1. Wildfire Impacts and Trends

Results from our analysis showed a clear increase in the area burned by wildfires across California during the 21-year study period. These results are consistent with the overall trends in wildfire activity that have been reported across the United States [31,32,33]. These results are also reasonable given the buildup of dry vegetation over time compounded by climatic changes that have resulted in higher spring and summer temperatures, reduced snowpack, and earlier spring snowmelt, which increase moisture stress on vegetation and render forests more fire-prone even at high elevation [4,9,10,25]. According to CAL FIRE, climate change has enabled particularly dry vegetation that allows wildfires to spread more aggressively, as well as periods of heavier-than-usual rainfall which allows heavy vegetative growth and greater amounts of fuel for potential wildfires [9].

We identified a doubling of the number of Census tracts that experienced major wildfire burning over the 21-year study period. This trend was most apparent when considering wildfires of all sizes, but it was also evident when only considering major (>100 acres) wildfires. Similarly, this analysis identified a near doubling of the number of people residing in Census tracts that experienced wildfire. Since we did not examine changes in population statistics over time, this pattern can be attributed to the expansion of wildfire burned area as opposed to population growth. Such residents and those living close to these areas may be adversely affected by wildfire in a number of ways, including physical and psychological stress from evacuation, property loss, physical injury or death, and smoke-related impacts to health [12,13]. While this study did not directly measure these impacts, the increasing number of Census tracts (and associated residents) experiencing wildfire over time suggests the likely increased occurrence of these impacts over time. While previous studies reported wildfire trends over time, these findings help to showcase how such trends have translated to human impacts as opposed to only ecological impacts and burned area.

### 4.2. Census Tract Analysis

Our analysis showed that rural Census tracts on average experienced a much higher degree of wildfire burning compared to urban Census tracts, and that such Census tracts also had higher rates of poverty, unemployment, and vacant housing, as well as higher proportions of low-income residents, residents without college degrees, and households without a computer and/or internet. This finding underscores a potential issue of environmental justice in which disadvantaged families who cannot afford to live in urban areas are rendered at greater risk of dangerous wildfires that may impact their health and further exacerbate socioeconomic inequities.

When dividing Census tracts according to urban and rural land designations and assessing socioeconomic factors, we found that Census tracts with a higher fire frequency and burned area had lower proportions of minority group populations. An important exception was for Native Americans, which tended to be in greater proportion in Census tracts more affected by wildfire. This pattern was observable across both urban and rural Census tracts. These findings are reasonable given that minority populations in the US tend to be most prevalent in populated cities and other more heavily urbanized areas, which are less prone to wildfires [34]. In contrast, Native American populations tend to reside in more rural landscapes that are more prone to wildfires [35]. Although our analysis separated urban and rural Census tracts, differences in urbanization still exist within urban and rural designations that cannot be captured by a binary category. For instance, some rural areas can be more or less rural than others, and the same is true for urban designations.

Although Census tracts with greater proportions of minority group populations tended to experience lower wildfire frequencies and fractions of burn area, such groups may nonetheless be at high risk of wildfires impacts due to socioeconomic barriers, risks which may defer between subgroups. While limited literature exists to identify which subgroup is at greatest risk, key factors can nonetheless help to identify elevated risk among minority populations. Communities with greater language barriers, including Hispanic and indigenous residents, are more likely to face challenges as it relates to heeding evacuation warnings and health guidelines (e.g., tips to reducing smoke exposure) that are disseminated in English, as well as maintaining comfort among health providers [14].

Additionally, research has shown undocumented residents within Hispanic and indigenous immigrant communities to be marginalized and disproportionately underserved both before and after wildfire disasters [36,37]. Méndez et al. (2020) pointed out, for instance, that such communities are disproportionately affected by racial discrimination, exploitation, economic hardship, and fear of deportation, and that the emergency response and recovery efforts that occurred in the wake of the Thomas Fire in California (as an example) largely ignored the needs of such communities, with resources instead being directed toward more privileged individuals [36].

Another key finding in our study was that Census tracts with a higher fire frequency and greater proportions of burned area had substantially higher proportions of older (age > 65) residents. This is an important finding given that it may be more difficult for such residents to expeditiously evacuate during a wildfire, and because this age group is particularly vulnerable to health-related impacts of short-term wildfire smoke exposure [38,39,40] given their higher prevalence of pre-existing lung and heart diseases and due to important health-protective physiologic processes that decline with age [38].

Another key finding consistent between assessments of both wildfire frequency and burned area was that social and economic variables tended to exhibit stronger correlations with wildfire impacts when assessing urban, as opposed to rural, Census tracts. This difference may reflect stronger associations between socioeconomic factors and rural greenspace (where more vegetation/fuel exists) in urban areas compared to rural areas where greenspace/vegetation is a more common feature.

In general, Census tracts with higher proportions of low-income residents and lower proportions of high-income residents, as well as lower median household incomes and home values, experienced higher wildfire frequency. This may suggest differences in land management and/or the availability of resources for combatting wildfires before they grow large. Interestingly, the opposite pattern was evident when examining wildfire burn area, where more affluent Census tracts tended to experience the greatest burning as a fraction of total area. This pattern was less pronounced for rural Census tracts. This urban/rural difference may again reflect wealthier neighborhoods being located in more vegetated, fire-prone WUI areas on the outskirts of town within urban Census tracts, whereas rural areas may exhibit less of a socioeconomic gradient as it relates to the degree of ruralness. This conclusion is supported by an analysis by Wigtil et al. (2016), which assessed wildfire risk and vulnerability across the US and found that higher-income areas were generally at greater risk to wildfires [27].

Findings were more mixed when assessing other vulnerability-related characteristics. For instance, Census tracts that experienced a higher fire frequency over the 21-year study also had higher proportions of residents/households without health coverage, a college education, occupancy, internet, and computers, suggesting an overlap between wildfire impacts and wildfire vulnerability. However, when examining these characteristics in the context of the percentage of land area burned (rather than fire frequency), correlations were more modest and, in some cases, opposite.

Given disparate findings observed when assessing fire frequency versus burned area, it is important to discuss the potential utility of each metric in terms of explaining wildfire impacts. When considering fire frequency, this study was able to identify Census tracts that are repeatedly burdened by wildfire on an inter-annual basis. This could arise from differences in population density, inhabitation of WUI areas, or land/vegetation management. However, this metric does not enable an understanding as to the severity of impacts. The severity of wildfire burning over the study period can instead be understood through our assessment of the proportion of land area burned. Despite the utility of this latter metric, this metric does not enable an understanding as to the recurrence of wildfires within a Census tract.

Given an understanding of the advantages and limitations of each wildfire impact metric, we can better infer the implications of our results. Regarding the positive correlation between wildfire vulnerability indicators and fire frequency, this may be the result of differences in vegetation management between high- and low-income Census tracts and/or the availability of resources for combating wildfires, as noted earlier.

General strategies that can help reduce the human health impacts related to wildfires include the establishment of evidence-based evacuation plans, as well as greater education to help residents reduce their exposure to wildfire smoke. Effective strategies to reduce smoke exposure during wildfire events include staying indoors, closing the doors and windows, and using an indoor air purification device [41,42,43]. Such interventions can reduce air pollution concentrations by over 90% [41]. During evacuation, measures can also be taken to help reduce psychological stress, especially stress caused to elderly residence with language and cultural barriers. For instance, Asfaw et al. (2020) showed that indigenous elderly residents had negative experiences with health services during the Sandy Lake First Nation Wildfire evacuation due to accommodation-related challenges, language barriers, and lack of access to traditional food, which can be overcome in the future with better designed action plans that take such needs into account. Additionally, research on post-disaster recovery efforts highlights the importance of “community strength and support” systems among communities affected by wildfire [37]. Collectively, these measures will likely be increasingly important to consider in the future as climatic extremes lead to the exacerbation of wildfire events and the occurrence of so-called “compound hazards” that may strain preventative measures, rescue efforts, and municipal resources [44].

### 4.3. Strengths and Limitations

A strength of this study is the comprehensive analysis of burned area over an extended 21-year period, which enabled us to understand how wildfire activity has changed over time. Another strength is the assessment of socioeconomic correlations at a high spatial resolution (Census tract level), which allowed us to identify potential health- and risk-related inequities. Moreover, conducting separate analyses according to both fire frequency and percentage area burned, as well as by rural and urban land designations, provided important comparisons that allowed for more robust conclusions. A final strength of this study is the use of nationally reported and publicly available Census and ACS data, as well as wildfire burn perimeters, which enables this study approach to be employed across other states and regions in the US.

This study also includes limitations. For instance, findings in this analysis represent only an estimate of the annual number of residents affected by wildfire as this analysis did not take into account the impacts of wildfire smoke, evacuation, or injury, nor did it consider impacts to communities in Census tracts that were adjacent to, but not overlapping with, wildfires. Another limitation is the potential for temporal mismatch given that a single 5-year averaged ACS dataset was used to represent the entire study period. Lastly, this study employed correlation analyses. While this enabled us to understand which communities and demographics experienced the greatest wildfire burning and fire frequency, as well as ascertain whether or not patterns existed as it relates to social and economic factors, the approach is distinct from a multivariate regression analysis aimed at understanding causative associations. Therefore, while the present study was able to identify correlations and patterns, it cannot be said to demonstrate the causation that underlies these patterns. Future research aimed at examining various wildfire-related variables such as lightning strikes, temperature, and humidity, as well as land-use changes, land management, and access to resources in the context of socioeconomic characteristics, is needed to better understand the underlying causes that might explain the disparities outlined in this study.

## 5. Conclusions

This study examined wildfire-burned area over the entire state of California from the years 2000 to 2020 to understand how wildfire activity has changed over time and to identify whether burned area and frequency differed across Census tracts according to socioeconomic characteristics over the study period. Results showed a clear increase in the area burned by wildfires across California over time, with a doubling in the number of Census tracts that experienced major wildfires and a near doubling of the number of people residing in wildfire-impacted Census tracts. Although rural Census tracts are intuitively more prone to wildfires, our analysis showed that such areas not only sustained three times more wildfire on average, but also tended to be characterized by higher rates of poverty, unemployment, and vacant housing, as well as higher proportions of low-income residents and residents without college degrees. Census tracts with a higher fire frequency and burned area had lower proportions of minority groups on average, except when considering Native American populations, of which a greater proportion resided in highly impacted Census tracts. Another key finding was that Census tracts most impacted by fire tended to have substantially greater proportions of elderly residents, which is of high concern given the disproportionate vulnerability of this population to smoke and other fire-related impacts. Collectively, these findings underscore important issues of environmental injustice among physically and socioeconomically vulnerable communities, in turn highlighting the need for more focused aid and recovery efforts on the part of municipalities and other fire-prevention and relief entities, as well as policymakers and state agencies who help determine the allocation of resources before, during, and after wildfires in the state of California.

## Data Availability

Publicly available datasets were analyzed in this study. Wildfire data can be found here: Historic Perimeter Combined 2000–2018 (https://data-nifc.opendata.arcgis.com/datasets/historic-perimeters-combined-2000–2018?geometry=24.338%2C-9.831%2C91.487%2C74.202, accessed on 2 April 2021), Historic Perimeters 2019 (https://data-nifc.opendata.arcgis.com/datasets/historic-perimeters-2019?geometry=133.339%2C27.667%2C-13.086%2C66.646, accessed on 2 April 2021), and Operational Data Archive 2020 (https://data-nifc.opendata.arcgis.com/datasets/operational-data-archive-2020, accessed on 2 April 2021), while ACS data for social, economic, housing, and other demographic factors can be found here: https://data.census.gov/cedsci/all?g=0400000US06,06.140000&d=ACS%205-Year%20Estimates%20Data%20Profiles, accessed on 2 April 2021.

## Supplementary Materials

The following are available online at https://www.mdpi.com/article/10.3390/ijerph18083921/s1: Figure S1. Satellite-rendered image of Thomas Fire as of 7 December 2017, alongside map showing total burn area caused by this fire; Table S1. Correlation matrix showing the temporal relationship between annual wildfire statistics from 2000 to 2021 in California; Table S2. Average of socioeconomic variables across urban Census tracts grouped according to fire frequency from 2000–2020; Table S3. Average of socioeconomic variables across rural Census tracts grouped according to fire frequency from 2000–2020; Table S4. Average of socioeconomic variables across urban Census tracts grouped by the percentage of cumulative Census tract land area burned from 2000–2020; Table S5. Average of socioeconomic variables across rural Census tracts grouped by the percentage of cumulative Census tract land area burned from 2000–2020.

## References

[1] Smith A.B. (2020). 2010–2019: A Landmark Decade of U.S. Billion-Dollar Weather and Climate Disasters.

[2] Smith A., Lott N., Houston T., Shein K., Crouch J., Enloe J.U.S. (2020). Billion-Dollar Weather & Climate Disasters 1980–2020.

[3] DIttrich R., McCallum S. (2020). How to measure the economic health cost of wildfires-A systematic review of the literature for northern America. Int. J. Wildland Fire.

[4] Westerling A.L., Hidalgo H.G., Cayan D.R., Swetnam T.W. (2006). Warming and earlier spring increase western U.S. forest wildfire activity. Science.

[5] Oregon Department of Forestry (ODF) (2020). ODF Fire History 1911–2020.

[6] California Department of Forestry and Fire Protection (2020). Top 20 Largest California Wildfires.

[7] California Department of Forestry and Fire Protection (2020). Top 20 Most Destructive California Wildfires.

[8] California Department of Forestry and Fire Protection (2018). Top 20 Deadliest California Wildfires.

[9] California Department of Forestry and Fire Protection (2019). Community Wildfire Prevention & Mitigation Report.

[10] NOAA Climate at a Glance. https://www.ncdc.noaa.gov/cag/statewide/time-series/.

[11] Agee J.K., Lolley M.R. (2006). Thinning and prescribed fire effects on fuels and potential fire behavior in an eastern cascades forest. Fire Ecol..

[12] McDermott B.M., Lee E.M., Judd M., Gibbon P. (2005). Posttraumatic Stress Disorder and General Psychopathology in Children and Adolescents Following a Wildfire Disaster. Can. J. Psychiatry.

[13] Reid C.E., Brauer M., Johnston F.H., Jerrett M., Balmes J.R., Elliott C.T. (2016). Critical review of health impacts of wildfire smoke exposure. Environ. Health Perspect..

[14] Asfaw H.W., McGee T.K., Christianson A.C. (2020). Indigenous Elders’ Experiences, Vulnerabilities and Coping during Hazard Evacuation: The Case of the 2011 Sandy Lake First Nation Wildfire Evacuation. Soc. Nat. Resour..

[15] Chen H., Samet J.M., Bromberg P.A., Tong H. (2021). Cardiovascular health impacts of wildfire smoke exposure. Part. Fibre Toxicol..

[16] Gan R.W., Liu J., Ford B., O’Dell K., Vaidyanathan A., Wilson A., Volckens J., Pfister G., Fischer E.V., Pierce J.R. (2020). The association between wildfire smoke exposure and asthma-specific medical care utilization in Oregon during the 2013 wildfire season. J. Expo. Sci. Environ. Epidemiol..

[17] Wu J., Winer A., Delfino R. (2006). Exposure assessment of particulate matter air pollution before, during, and after the 2003 Southern California wildfires. Atmos. Environ..

[18] Vedal S., Dutton S.J. (2006). Wildfire air pollution and daily mortality in a large urban area. Environ. Res..

[19] Aguilera R., Gershunov A., Ilango S.D., Morales J.G. (2019). Santa Ana Winds of Southern California Impact PM2.5 with and without Smoke from Wildfires. GeoHealth.

[20] Cleland S.E., West J.J., Jia Y., Reid S., Raffuse S., O’Neill S., Serre M.L. (2020). Estimating Wildfire Smoke Concentrations during the October 2017 California Fires through BME Space/Time Data Fusion of Observed, Modeled, and Satellite-Derived PM2.5. Environ. Sci. Technol..

[21] Agyapong V.I.O., Ritchie A., Brown M.R.G., Noble S., Mankowsi M., Denga E., Nwaka B., Akinjise I., Corbett S.E., Moosavi S. (2020). Long-Term Mental Health Effects of a Devastating Wild fire Are Amplified by Socio-Demographic and Clinical Antecedents in Elementary and High School Staff. Front. Psychiatry.

[22] Stewart S.I., Radeloff V.C., Hammer R.B., Hawbaker T.J. (2007). Defining the Wildland—Urban Interface. J. For..

[23] Faivre N., Commission E., Jin Y., Goulden M., Randerson J. (2014). Controls on the spatial pattern of wildfire ignitions in Southern California Controls on the spatial pattern of wildfire ignitions in Southern California. Int. J. Wildland Fire.

[24] Gabbe C.J., Pierce G., Oxlaj E. (2020). Subsidized Households and Wildfire Hazards in California. Environ. Manag..

[25] Radeloff V.C., Helmers D.P., Anu Kramer H., Mockrin M.H., Alexandre P.M., Bar-Massada A., Butsic V., Hawbaker T.J., Martinuzzi S., Syphard A.D. (2018). Rapid growth of the US wildland-urban interface raises wildfire risk. Proc. Natl. Acad. Sci. USA.

[26] Davies I.P., Haugo R.D., Robertson J.C., Levin P.S. (2018). The unequal vulnerability of communities of color to wildfire. PLoS ONE.

[27] Wigtil G., Hammer R.B., Kline J.D., Mockrin M.H., Stewart S.I., Roper D., Radeloff V.C. (2016). Places where wildfire potential and social vulnerability coincide in the coterminous United States. Int. J. Wildland Fire.

[28] Adams M.D.O., Charnley S. (2020). The Environmental Justice Implications of Managing Hazardous Fuels on Federal Forest Lands. Ann. Am. Assoc. Geogr..

[29] United States Department of Agriculture (2017). National Infrared Operations/NIROPS Hi-Tech Heat-Seekers.

[30] Hart G., Cromartie J., Morrill R. Rural-Urban Commuting Area Codes (RUCAs). https://depts.washington.edu/uwruca/ruca-data.php.

[31] Greskowiak J., Brewer S., Arnold J., Mortiz M. (2014). Large wildfire trends in the western United States, 1984–2011. Geophys. Res. Lett..

[32] Patel K. Six Trends to Know about Fire Season in the Western U.S.. https://climate.nasa.gov/blog/2830/six-trends-to-know-about-fire-season-in-the-western-us/.

[33] Miller J.D., Safford H. (2012). Trends in wildfire severity: 1984 to 2010 in the Sierra Nevada, Modoc Plateau, and southern Cascades, California, USA. Fire Ecol..

[34] Parker K., Juliana H., Brown A., Fry R., Cohn D., Igielnik R. (2018). What Unites and Divides Urban, Suburban and Rural Communities.

[35] Dewees S., Marks B. (2017). Twice Invisible: Understanding Rural Native America.

[36] Méndez M., Flores-Haro G., Zucker L. (2020). The (in)visible victims of disaster: Understanding the vulnerability of undocumented Latino/a and indigenous immigrants. Geoforum.

[37] Domínguez D., Yeh C. (2020). Social justice disaster relief, counseling, and advocacy: The case of the Northern California wildfires. Couns. Psychol. Q..

[38] Orr A., Migliaccio C.A.L., Buford M., Ballou S., Migliaccio C.T. (2020). Sustained effects on lung function in community members following exposure to hazardous pm2.5 levels from wildfire smoke. Toxics.

[39] Jones C.G., Rappold A.G., Vargo J., Cascio W.E., Kharrazi M., McNally B., Hoshiko S. (2020). Out-of-Hospital Cardiac Arrests and Wildfire-Related Particulate Matter During 2015–2017 California Wildfires. J. Am. Heart Assoc..

[40] United States Environmental Protection Agency (USEPA) (2009). Integrated Science Assessment for Particulate Matter.

[41] Stauffer D., Autenrieth D.A., Hard J.F., Capoccia S. (2020). Control of wildfire-sourced PM2.5 in an office setting using a commercially available portable air cleaner. J. Occup. Environ. Hyg..

[42] Xiang J., Huang C.-H., Shirai J., Liu Y., Carmona N., Zuidema C., Austin E., Gould T., Larsona T., Seto E. (2021). Field measurements of PM2.5 infiltration factor and portable air cleaner effectiveness during wildfire episodes in US residences. Sci. Total Environ..

[43] Frisk W.J., Chan W.J. (2017). Health benefits and costs of filtration interventions that reduce indoor exposure to PM2.5 during wildfires. Indoor Air.

[44] Aghakouchak A., Chiang F., Huning L.S., Love C.A., Mallakpour I., Mazdiyasni O., Moftakhari H., Papalexiou S.M., Ragno E., Sadegh M. (2020). Climate Extremes and Compound Hazards in a Warming World. Annu. Rev. Earth Planet. Sci..

