# Supplementary material for: Disproportionate Impacts of Wildfires among Elderly and Low-Income Communities in California from 2000–2020
Source: Int J Environ Res Public Health. 2021 Apr 8;18(8):3921. doi: 10.3390/ijerph18083921 (PMC8068328; doi:10.3390/ijerph18083921)
Supplement: Supplementary file 1 [file ijerph-18-03921-s001.pdf]

International Journal of
Environmental Research and Public Health

MDPI

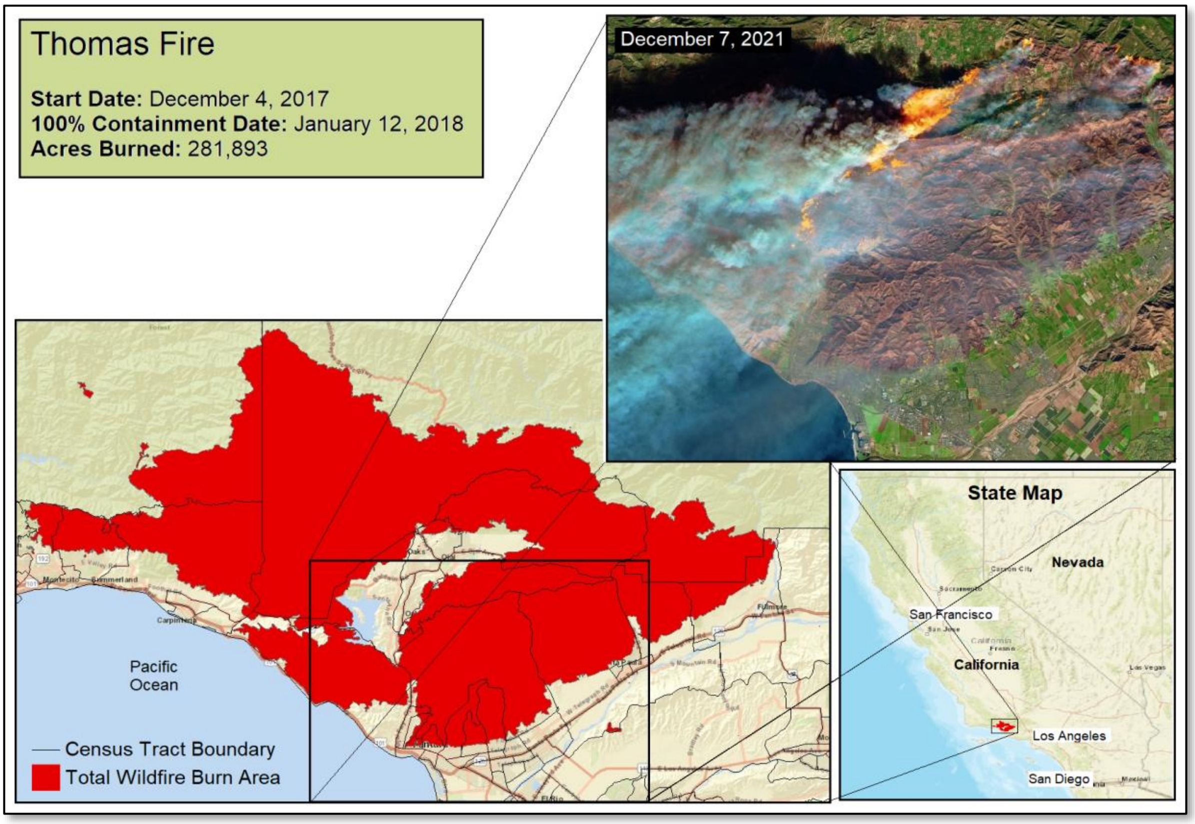

Figure S1. Satellite-rendered image of Thomas Fire as of December 7th, 2017, alongside map showing total burn area caused by this fire. The Thomas Fire was the largest wildfire on record in California as of the date of the event. Satellite Image Source: NASA Earth Observatory. Satellite Image Description: Derived from MODIS satellite data with false-color post-processing performed by the European Space Agency.

Table S1. Correlation matrix showing the temporal relationship between annual wildfire statistics from 2000 to 2021 in California.

**Correlation Matrix (n=21)**		**Avg. Fire Size**	**Total Acres Burned**	**# of CTs burned annually by fire size category (1000 acres)**					**# of CTs by annual area burned (%)**				**Population within CTs by annual area burned (%)**					**# of Fires by size category (1000 acres)**	
				>0	>0.1	>1	>10	>100	>10	>25	>50	>75	>0	>10	>25	>50	>75	>0	>1
Avg. Fire Size		**1.00**	**0.93**	-0.09	0.09	**0.45**	**0.77**	**0.89**	0.41	0.34	0.20	0.08	-0.13	0.30	0.25	0.12	0.05	–0.32	0.15
Total Acres Burned			**1.00**	0.23	0.37	**0.69**	**0.93**	**0.96**	**0.64**	**0.57**	**0.44**	0.36	0.19	**0.53**	**0.49**	0.37	0.33	**–0.02**	–0.40
# of CTs burned annually by fire size category (1,000 acres)	>0			**1.00**	**0.93**	**0.79**	**0.49**	0.19	**0.80**	**0.78**	**0.79**	**0.79**	**0.99**	**0.81**	**0.80**	**0.80**	**0.81**	**0.85**	**0.63**
	>0.1				**1.00**	**0.90**	**0.61**	0.30	**0.89**	**0.87**	**0.87**	**0.83**	**0.91**	**0.89**	**0.88**	**0.87**	**0.85**	**0.70**	**0.68**
	>1					**1.00**	**0.87**	**0.62**	**0.95**	**0.91**	**0.87**	**0.81**	**0.76**	**0.91**	**0.90**	**0.85**	**0.82**	**0.50**	**0.65**
	>10						**1.00**	**0.88**	**0.81**	**0.75**	**0.66**	**0.58**	**0.46**	**0.73**	**0.71**	**0.61**	**0.57**	0.22	**0.58**
	>100							**1.00**	**0.57**	**0.49**	0.37	0.32	0.15	**0.45**	0.40	0.28	0.28	0.00	**0.44**
# of CTs by annual area burned (%)	>10%								**1.00**	**0.98**	**0.94**	**0.87**	**0.80**	**0.98**	**0.96**	**0.91**	**0.87**	**0.44**	**0.51**
	>25%									**1.00**	**0.98**	**0.93**	**0.79**	**0.97**	**0.99**	**0.96**	**0.93**	0.38	0.41
	>50%										**1.00**	**0.97**	**0.80**	**0.94**	**0.99**	**0.99**	**0.97**	0.41	0.39

	>75 %	**1.00**	**0.81**	**0.87**	**0.94**	**0.95**	**0.99**	**0.45**	0.38
	>0 %		**1.00**	**0.82**	**0.82**	**0.82**	**0.83**	**0.83**	**0.57**
	>10 %			**1.00**	**0.98**	**0.95**	**0.88**	**0.46**	**0.48**
Population within CTs by annual area burned (%)	>25 %				**1.00**	**0.98**	**0.95**	0.42	0.42
	>50 %					**1.00**	**0.97**	**0.44**	0.40
	>75 %						**1.00**	**0.49**	0.42
# of Fires by size category (1000 acres)	>0							**1.00**	**0.71**
	>1								**1.00**

Bold font indicates statistical significance ($p < 0.05$).

Table S2. Average of socioeconomic variables across urban Census tracts grouped according to fire frequency from 2000–2020.

**Socioeconomic Variable**	**Fire Frequency**																				
	**0**	**1**	**2**	**3**	**4**	**5**	**6**	**7**	**8**	**9**	**10**	**11**	**12**	**13**	**14**	**15**	**16**	**17**	**18**	**19**	**20**
% Population (Asian)	15.3	9.0	11.1	11.5	7.2	8.6	7.5	7.9	4.3	3.3	7.1	3.3	3.1	4.9	4.3	0.0	1.3	4.8	1.5	4.3	2.2
% Population (Hispanic)	1.2	2.3	7.7	0.5	19.1	0.8	0.8	0.6	1.6	0.7	0.6	0.9	0.6	0.9	1.5	0.4	0.9	0.8	0.9	0.5	0.3
% Population (Native Am.)	0.7	0.6	1.1	0.8	0.7	1.1	0.5	0.8	1.2	2.0	1.0	1.2	1.0	2.5	6.5	0.6	1.0	2.8	3.1	0.4	19.8
% Population (African Am.)	6.4	3.8	3.2	3.4	3.9	3.2	2.2	2.6	3.1	1.5	1.7	1.9	2.6	0.1	2.6	5.0	0.8	3.6	1.3	3.0	1.4
% Vacant Housing Units	6.1	6.4	7.9	8.9	10.2	9.9	16.9	14.1	17.2	22.0	19.4	21.9	11.0	33.9	16.4	39.5	20.4	26.4	28.5	24.2	43.3
% Unemployed	6.9	6.0	5.8	5.9	6.2	5.5	4.7	6.5	6.7	6.9	5.6	6.8	5.1	16.0	7.7	5.3	6.8	7.0	8.3	6.7	7.9
% without Health Coverage	8.8	6.0	5.4	5.5	6.5	5.8	5.2	6.4	7.9	8.2	5.2	7.1	6.7	13.2	11.3	11.9	6.4	6.8	9.7	5.6	9.4
% Poverty	11.5	6.4	5.8	6.3	6.9	6.1	5.0	6.5	7.7	7.7	4.4	10.6	5.0	11.1	14.7	6.3	6.4	6.6	11.4	11.4	14.1
Median Household Income ($100K)	0.8	1.0	1.0	1.0	1.0	1.1	1.0	0.9	0.8	0.9	1.0	0.7	0.9	0.6	0.7	0.5	0.7	0.7	0.6	0.6	0.4
Median Home Value ($100K)	5.4	5.6	6.2	6.1	5.7	5.6	6.1	5.8	5.5	4.6	5.6	5.4	3.9	2.6	3.8	2.3	3.5	3.6	2.5	2.6	2.5
% Low Income	26.5	17.9	17.8	20.0	17.9	16.6	16.6	20.4	22.7	22.8	15.0	27.1	24.1	35.7	28.5	33.1	28.1	24.0	32.1	42.4	40.5
% High Income	33.8	44.8	48.4	46.4	47.1	50.5	47.3	44.5	37.7	39.4	48.0	30.0	40.7	24.8	33.7	29.3	35.4	36.4	26.3	28.3	20.7
% population > 65 years old	19.7	23.0	24.2	23.4	23.8	22.2	25.8	26.4	22.4	28.5	25.1	23.9	25.3	25.7	20.1	19.1	29.0	26.0	25.0	29.6	37.3
% Households without Computer	8.7	5.8	5.4	6.6	6.7	5.4	7.1	7.3	8.7	7.7	3.7	8.9	9.5	14.8	10.9	6.4	13.2	10.4	16.3	12.3	24.9
% Households without Internet	16.2	11.2	10.3	11.7	12.4	10.5	12.8	13.3	14.1	14.2	10.1	18.3	14.5	24.7	19.1	11.8	25.7	17.3	25.8	22.5	37.5
% Households without College Degree	67.1	62.1	58.6	61.5	64.3	62.1	60.0	63.9	71.7	64.8	58.3	76.7	74.8	76.6	70.8	79.4	73.1	74.9	80.1	72.9	77.4
Number of Census Tracts (count)	6,684	332	172	83	60	32	19	22	22	13	9	7	5	3	6	1	6	6	3	2	1

Table S3. Average of socioeconomic variables across rural Census tracts grouped according to fire frequency from 2000–2020.

**Socioeconomic Variable**	**Fire Frequency**																				
	**0**	**1**	**2**	**3**	**4**	**5**	**6**	**7**	**8**	**9**	**10**	**11**	**12**	**13**	**14**	**15**	**16**	**17**	**18**	**19**	**20**
% Population (Asian)	3.2	2.3	1.5	1.4	2.2	1.8	2.6	1.9	1.4	1.6	2.2	1.9	0.8	0.5	1.9	1.4	1.1	1.3	1.4	2.8	1.1
% Population (Hispanic)	1.1	0.7	0.8	0.8	0.7	0.6	0.7	0.7	1.0	0.6	0.4	1.7	1.1	0.3	0.7	1.0	0.4	0.7	0.4	0.6	0.4
% Population (Native Am.)	1.3	2.0	1.9	3.3	1.5	2.2	1.5	1.7	6.6	3.8	2.3	2.9	5.7	7.5	4.0	1.6	13.8	4.6	2.6	6.1	4.3
% Population (African Am.)	3.3	1.3	1.3	2.9	2.1	1.1	1.8	1.5	1.1	2.0	1.0	1.7	0.5	0.6	0.9	2.5	0.5	0.6	2.4	0.8	0.5
% Vacant Housing Units	14.0	24.4	16.5	26.3	30.1	31.4	26.4	19.2	19.5	15.9	30.9	33.8	24.3	24.8	34.4	23.2	48.1	30.1	23.6	45.6	38.9
% Unemployed	9.2	7.7	7.6	12.4	8.3	7.7	8.3	9.2	9.1	9.7	7.9	10.1	9.5	9.8	9.9	10.6	8.3	7.8	10.5	7.6	11.6
% without Health Coverage	9.1	8.5	6.8	8.0	7.6	7.7	8.3	9.6	9.2	8.8	7.4	7.8	8.5	7.7	9.5	7.6	9.9	8.6	7.6	8.0	7.5
% Poverty	16.5	10.2	11.2	11.8	8.8	7.5	12.2	11.9	13.3	11.6	10.5	9.3	11.7	12.0	11.9	11.5	8.8	7.4	10.7	9.4	11.5
Median Household Income ($100K)	0.5	0.6	0.6	0.6	0.6	0.7	0.6	0.5	0.5	0.6	0.5	0.7	0.5	0.6	0.5	0.5	0.5	0.6	0.5	0.5	0.5
Median Home Value ($100K)	2.6	3.3	3.3	3.2	3.2	4.4	3.4	3.2	2.9	4.2	2.7	3.1	2.8	2.5	2.3	2.1	2.6	3.2	2.8	2.5	2.0
% Low Income	36.2	33.2	30.5	37.5	28.0	28.0	34.3	37.2	35.4	32.0	33.9	29.8	35.6	36.4	38.0	38.3	37.0	31.1	37.3	33.5	32.8
% High Income	20.2	23.7	25.3	23.4	26.1	31.0	23.6	19.6	22.1	26.1	20.7	27.9	18.6	20.8	19.7	20.7	18.9	24.6	15.2	19.3	21.0
% population > 65 years old	20.3	31.1	28.2	34.3	29.9	31.5	29.4	32.7	26.4	29.8	30.9	33.7	27.9	35.9	33.6	29.3	31.0	31.1	30.4	37.5	36.7

% Households without Computer	14.2	11.0	12.9	15.3	10.6	11.9	15.0	14.3	15.7	11.7	13.8	8.8	24.7	17.3	16.6	17.2	16.6	12.6	16.4	16.2	14.4
% Households without Internet	24.0	19.5	22.7	24.7	19.9	20.0	24.3	23.1	26.5	24.4	26.5	20.6	34.9	28.5	30.1	29.3	25.0	22.5	27.0	23.6	24.5
% Households without College Degree	82.1	74.6	78.5	76.0	74.9	69.7	77.1	76.5	80.6	76.0	79.5	77.7	83.5	81.4	82.5	83.5	76.2	79.8	80.9	76.0	73.9
Number of Census Tracts (count)	293	59	36	20	17	16	19	14	17	9	11	7	7	5	10	9	4	5	3	5	3

Table S4. Average of socioeconomic variables across urban Census tracts grouped by the percent of cumulative Census tract land area burned from 2000–2020.

Socioeconomic Variable	Area Burned (%)								
	0	0–10	10–20	20–30	30–40	40–50	50–60	60–70	70+
% Population (Asian)	15.3	8.7	8.8	7.9	10.7	9.7	10.3	8.3	9.7
% Population (Hispanic)	1.2	4.4	18.3	0.6	0.7	0.7	0.9	0.7	1.7
% Population (Native Am.)	0.7	0.8	0.8	1.0	0.8	2.2	1.1	1.3	0.9
% Population (African Am.)	6.4	3.7	3.5	2.4	4.6	3.1	4.0	2.4	2.6
% Vacant Housing Units	6.1	8.0	12.4	10.2	11.6	12.2	8.6	9.6	10.4
% Unemployed	6.9	6.2	6.5	5.3	6.0	6.0	5.9	6.7	5.2
% without Health Coverage	8.9	6.1	5.8	5.6	6.9	7.1	6.4	5.0	5.4
% Poverty	11.5	7.1	7.6	5.7	5.9	6.2	6.5	5.9	4.3
Median Household Income ($100K)	0.8	0.9	0.9	1.0	1.0	0.9	1.0	1.0	1.1
Median Home Value ($100K)	5.4	5.4	5.4	6.0	5.4	5.3	7.0	5.8	6.7
% Low Income	26.5	20.1	20.4	17.5	17.4	19.2	18.2	17.3	14.9
% High Income	33.8	43.2	43.1	47.5	45.3	43.9	46.8	49.1	52.1
% population > 65 years old	19.7	23.3	24.0	26.1	20.4	20.3	26.5	24.0	25.2
% Households without Computer	8.7	6.8	6.8	6.6	6.2	5.7	6.2	4.8	4.7
% Households without Internet	16.2	12.9	13.5	11.5	11.0	10.7	11.2	8.5	8.8
% Households without College Degree	67.1	64.3	64.0	60.7	62.5	64.8	58.6	60.0	56.1
Number of Census Tracts (count)	6,684	415	68	60	51	34	35	27	115

Table S5. Average of socioeconomic variables across rural Census tracts grouped by the percent of cumulative Census tract land area burned from 2000–2020.

Socioeconomic Variable	Area Burned (%)								
	0	0–10	10–20	20–30	30–40	40–50	50–60	60–70	70+
% Population (Asian)	3.2	1.9	1.7	1.1	3.4	1.6	2.5	1.2	1.4
% Population (Hispanic)	1.1	0.8	0.5	0.9	0.4	0.9	0.6	0.4	0.9
% Population (Native Am.)	1.3	2.4	6.8	1.5	2.1	3.9	4.5	2.8	3.2
% Population (African Am.)	3.3	1.7	0.9	2.3	1.5	1.9	0.3	0.7	0.7
% Vacant Housing Units	14.0	24.2	31.4	32.7	29.8	25.6	23.6	18.5	22.4
% Unemployed	9.2	8.7	9.0	9.5	8.7	8.9	6.3	7.2	10.1
% without Health Coverage	9.1	8.4	8.0	8.7	6.1	7.4	7.1	6.7	8.6
% Poverty	16.5	11.0	9.4	12.9	10.9	9.0	9.6	6.3	12.5
Median Household Income ($100K)	0.5	0.6	0.5	0.5	0.6	0.6	0.6	0.6	0.5
Median Home Value ($100K)	2.6	3.2	3.3	2.3	3.3	3.4	3.2	3.4	3.2
% Low Income	36.2	32.9	34.1	36.3	35.4	27.4	30.8	29.6	39.2
% High Income	20.2	24.0	23.3	21.8	23.3	28.9	25.0	23.2	18.1
% population > 65 years old	20.3	30.3	33.1	30.0	34.4	33.2	28.3	32.7	30.6
% Households without Computer	14.2	13.2	14.2	17.3	11.4	13.7	12.5	11.7	15.1
% Households without Internet	24.0	23.2	23.8	26.9	21.1	26.6	19.6	19.0	24.6
% Households without College Degree	82.1	76.9	75.3	79.9	76.5	77.3	76.9	76.2	79.0
Number of Census Tracts (count)	293	172	28	17	9	9	12	9	20
